# Personality and features of metacognition and perception of everyday life

**DOI:** 10.1192/j.eurpsy.2021.1180

**Published:** 2021-08-13

**Authors:** V. Lianguzova

**Affiliations:** Psychology, Moscow State University, Moscow, Russian Federation

**Keywords:** everyday life, metacognition, perception, Personality

## Abstract

**Introduction:**

In our study, we aimed to understand how an individual perceives everyday life, as well as, which role the features of metacognitions and personality play in this process. Everyone is immersed in society and therefore exposed to external influences. Attention has already been focused on the relationship between metacognition and social context. “Internal orientation” depends largely on our personal and family history, socio-economic situation, group membership, and cultural context.

**Objectives:**

The sample consisted of 30 participants (women and men, M=25,7, Sd=3,6), selected by the criterion of personal interest in this research.

**Methods:**

The study consisted of several stages. The first stage was devoted to the theoretical analysis of everyday life in modern psychology. The data were processed using descriptive qualitative analysis using the phenomenological method, where we identified categories (Central topics) among the participants ‘ responses. To study the features of metacognition, we selected methods for assessing metacognitive involvement in awareness of internal activity, as well as metacognitive beliefs. The metacognitive awareness inventory questionnaire (MAIL) (Schraw & Dennison, 1994) in adaptation (Karpov & Skiteva, 2005) allows you to assess the level of metacognitive engagement, answer the question about the level of metacognitive awareness of the participant.

**Results:**

Pearson correlation is revealed a significant relationship between MAI and cognitive self-consciousness (p=0.003), positive beliefs (p=0.002), and needs of controling the thoughts (p=0.076).

**Conclusions:**

Our research opens the study of the subjective dimensions of person-situation-activity and offers a way of linking research on personality with research on the social processes whereby persons conduct their everyday lives.

